# Land–atmosphere–ocean coupling associated with the Tibetan Plateau and its climate impacts

**DOI:** 10.1093/nsr/nwaa011

**Published:** 2020-02-06

**Authors:** Yimin Liu, Mengmeng Lu, Haijun Yang, Anmin Duan, Bian He, Song Yang, Guoxiong Wu

**Affiliations:** 1 State Key Laboratory of Numerical Modeling for Atmospheric Sciences and Geophysical Fluid Dynamics, Institute of Atmospheric Physics, Chinese Academy of Sciences (CAS), Beijing 100029, China; 2 College of Earth Science, University of Chinese Academy of Sciences, Beijing 100049, China; 3 CAS Center for Excellence in Tibetan Plateau Earth Sciences, Beijing 100101, China; 4 School of Atmospheric Sciences & Guangdong Province Key Laboratory for Climate Change and Natural Disaster Studies, Sun Yat-sen University, Guangzhou 510275, China; 5 Department of Earth and Planetary Sciences, Harvard University, Cambridge, MA 02138, USA; 6 Laboratory for Climate and Ocean-Atmosphere Studies (LaCOAS) and Department of Atmospheric and Oceanic Sciences, School of Physics, Peking University, Beijing 100871, China; 7 Southern Marine Science and Engineering Guangdong Laboratory (Zhuhai), Zhuhai 519080, China

**Keywords:** Tibetan and Iranian Plateau system, atmospheric heat source, Asian summer monsoon, upstream climate, Atlantic Meridional Overturning Circulation

## Abstract

This paper reviews recent advances regarding land–atmosphere–ocean coupling associated with the Tibetan Plateau (TP) and its climatic impacts. Thermal forcing over the TP interacts strongly with that over the Iranian Plateau, forming a coupled heating system that elevates the tropopause, generates a monsoonal meridional circulation over South Asia and creates conditions of large-scale ascent favorable for Asian summer monsoon development. TP heating leads to intensification and westward extension (northward movement) of the South Asian High (Atlantic Intertropical Convergence Zone), and exerts strong impacts on upstream climate variations from North Atlantic to West Asia. It also affects oceanic circulation and buoyancy fields via atmospheric stationary wave trains and air–sea interaction processes, contributing to formation of the Atlantic Meridional Overturning Circulation. The TP thermal state and atmospheric–oceanic conditions are highly interactive and Asian summer monsoon variability is controlled synergistically by internal TP variability and external forcing factors.

## INTRODUCTION

The Tibetan Plateau (TP), which possesses a variety of complex landscapes, has average elevation of 4 km and its highest peak (Qomolangma; Everest) extends into the upper troposphere. The TP covers approximately 2.5 × 10^6^ km^2^, that is approximately 25% of China's land area, and constitutes the largest plateau on earth. Located in the subtropics of the eastern Afro-Eurasian continent, its longitudinal–latitudinal position, elevation, size and steep slopes (to the south and east) are the reasons for the considerable importance of the TP regarding the global climate. Its dynamic blocking effect in winter leads to division of the impinging westerly flow into northern and southern branches, which merge on the lee side of the plateau to form the strong East Asian jet stream downstream [1,2]. The pioneering studies of Yeh *et al*. [3] and Flohn [4] demonstrated that the TP is an atmospheric heat sink in winter but a heat source in summer. They initiated a new era of investigation of plateau meteorology, and the book *Meteorology of the Qinghai-Xizang Plateau* [5] summarizes many related achievements from subsequent studies.

Owing to the rapid progress of observational tools, computer technology, and numerical methods since the 1980s, the dynamics of the TP climate have been studied comprehensively, and in-depth investigations have revealed many features of the variation of the TP climate and of the climatic impact of the plateau on various timescales. Previous studies have mainly concentrated on the impact of the TP on the climate in Asia. In particular, the impact of the TP on the formation, intensity and variability of the Asian summer monsoon (ASM) has been investigated intensively [6–15]. It has also been reported recently that the TP strongly affects the climate in its ‘upstream’ (relative to the prevalent westerly flow) regions [16–19]. The influence of the TP on the ASM system is complex, and challenges and controversies regarding potential mechanisms have prompted discussions that have improved the overall understanding. One such example is the debate on the relative importance of TP thermal forcing versus mechanical forcing in maintaining the South Asian summer monsoon, which is reviewed here.

The effect of TP orography on oceanic properties emerged as an important issue in recent years in relation to the understanding of the formation of modern oceanic circulations [20–24]. Studies have shown that the modern-state thermohaline circulation, that is deep-water formation in the North Atlantic, is forced by the present-day distribution of high mountains, whereas it would occur in the Pacific if there were no mountains [25,26]. Freshwater transport is known as a major driver of this switch. Reduction of water vapor transport from the Pacific to the Atlantic because of high mountains contributes to increased (decreased) salinity and enhanced (reduced) deep-water formation in the North Atlantic (Pacific) [23,25]. However, it remains unknown which mountains play the most important role in shaping the modern oceanic circulation and the transient response of the oceanic circulation to orographic forcing remains unclear.

To date, most related studies have assumed a heat source over the TP and subsequently have investigated its influence on atmospheric circulation. However, the climate system is a fully coupled system and thus multisphere interactions must affect the heat source over the TP. This prompts questions concerning how the thermal status of the TP and of the oceans might interact through the atmospheric circulation, and how the TP and oceans might synergistically influence regional and global climate. Here, recent advances in the understanding of both the variations of the atmospheric heat source/sink (AHS) over the TP and the influences of the TP on the atmospheric circulation (e.g. the ASM and ‘upstream climate’ over West Asia, North Africa, Europe and North Atlantic) and the Atlantic Meridional Overturning Circulation (AMOC) are reviewed. Moreover, the feedback of the oceans on the AHS over the TP and the synergistic impact of the TP and ocean forcing on the ASM are considered.

## AHS OVER THE TP AND ASSOCIATED CIRCULATION

### TP heating and elevation dependence

**Figure 1.**
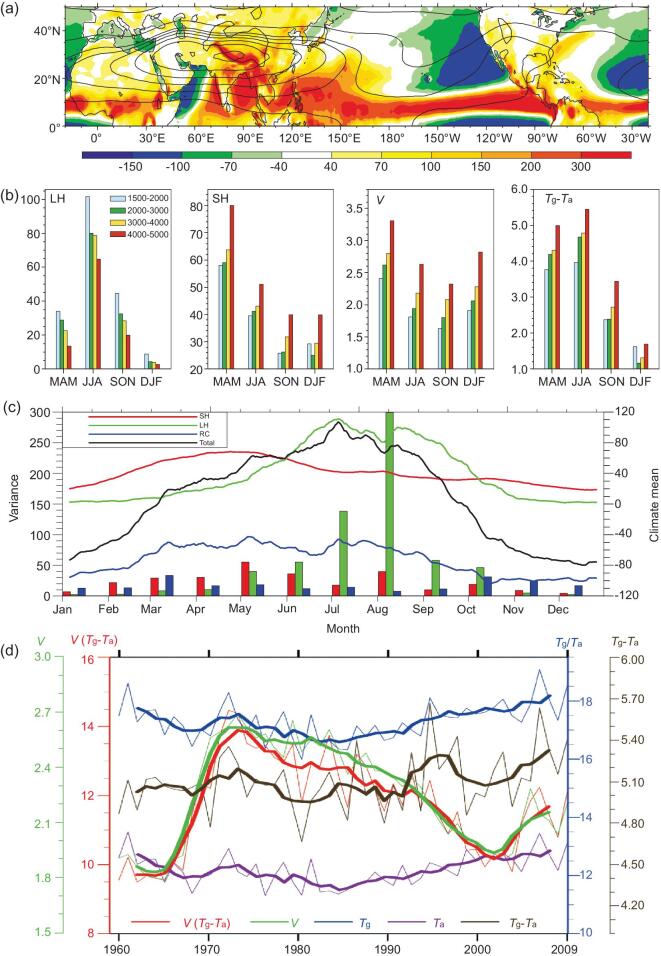
Atmospheric heating over the TP and its variation. (a) July mean from reanalysis data of geopotential height (contours, unit: dgpm) at 200 hPa and total diabatic heating (shading, unit: W m^−2^) [32]. (b) Elevation dependence of mean latent heat released to the atmosphere through condensation (LH, unit: W m^−2^), local surface sensible heat flux (SH, unit: W m^−2^), surface wind speed (*V*, unit: m s^−1^), and ground–air temperature difference (*T*_g_ − *T*_a_, unit: °C) for spring (MAM), summer (JJA), autumn (SON) and winter (DJF), averaged from 158 stations over the TP during 1979–2014. The blue, green, yellow and red bars are for areas with terrain height of 1.5–2.0, 2.0–3.0, 3.0–4.0 and 4.0–5.0 km, respectively. (c) Climatic mean (solid line; unit: W m^−2^) and monthly variance (bars) of each component of the TP heat source, as determined by averaged observational data from 73 stations. Red denotes SH, green denotes LH, blue denotes RC and black gives their sum [35]. (d) Evolutions of JJA means (averaged over the TP stations) of *T*_g_, *T*_a_, (*T*_g_ − *T*_a_), *V*, and the parameterized surface sensible heat flux *V* (*T*_g_ − *T*_a_). Heavy curves correspond to 11-year running mean [37].

For a given location and a given period, an AHS is defined as a net heat gain/loss:

$$\begin{equation*}{\rm AHS} = {\rm SH} + {\rm LH} + {\rm RC},\end{equation*}$$

where SH denotes surface sensible heat flux, LH is the latent heat released to the atmosphere associated with the change of water phase and RC is the convergence of the net radiation flux of the air column. Since the studies of Yeh *et al*. [3] and Flohn [4], considerable effort has been devoted to understanding the AHS over the TP and its impact on both weather and climate based on observations, process diagnosis, and numerical model experiments [5,27–31]. It has been revealed that the TP AHS is weakly negative in winter and strongly positive in summer. The July mean distributions of 200-hPa geopotential height and AHS over the TP are shown in Fig. 1a [32]. In addition to the strong tropical heating, a unique feature in the extratropics shown in Fig. 1a is the remarkable heating of >300 W m^−2^ over the southern slopes of the TP, which is comparable in magnitude to that associated with tropical convection. A continental-scale South Asian High (SAH) at 200 hPa can also be seen over Eurasia with its center located over the southwestern border of the TP.

Diabatic heating over the TP is dependent on elevation in all seasons [33]. LH reaches its maximum (minimum) in summer (winter) and it decreases with height in all seasons (Fig. 1b, left column) because the water vapor content at the lower elevated boundary is larger than that at the higher elevated boundary. Conversely, maximum SH is in spring and it increases with height throughout the year, except in winter when there is uneven snow accumulation over the TP (Fig. 1b, next to left column). The increase in SH with height is partially a result of the increasing wind speed (Fig. 1b, next to right column) and of the temperature difference between the land surface and surface air (*T*_g_ − *T*_a_) with elevation (Fig. 1b, right column). It is understood that the increase in SH with height is of significance in forcing the atmospheric circulation.

### Annual cycle and variability of the AHS over the TP

Owing to the complex conditions of the land surface, even state-of-the-art general circulation models (GCMs) and reanalysis products show large biases in reproducing the land surface processes over the TP [34]. The accumulation of routine meteorological records and field observation data has facilitated recent studies conducted to elucidate TP diabatic heating. Typically, SH is estimated using the bulk aerodynamic scheme and the total heat source is calculated by additionally considering precipitation and the radiant flux derived from satellite data. Zhao *et al*. [35] used the latest TP observations and satellite data to depict the monthly climatology of the annual cycle of various components of TP AHS and variance (Fig. 1c). It is shown that over the TP, SH dominates the AHS in spring, especially in March and April when SH (50–70 W m^−2^) is much larger than LH (5–20 W m^−2^). In May, the TP SH reaches its peak of the entire year. However, with the arrival of the rainy season, LH increases rapidly in May and June and it exceeds SH in late May and early June. Reaching its peak in July as the major component of the total heat source, LH surpasses SH throughout the summer and thus becomes the most important component of the AHS. The net radiant flux behaves as a cooling effect throughout the year, but the cooling effect in spring and summer (−40 to −70 W m^−2^) is smaller than in autumn and winter (−80 to −110 W m^−2^). The variance of SH in spring is larger than that of LH and the largest value is in May. The variance of LH in summer is larger than that of SH and it reaches a peak of 300 W^2^ m^−4^ in August. These results are consistent with the features revealed by station observations and reanalysis products (e.g. [33,36]).

Yu *et al*. [33] used the latest station observations to examine the temporal characteristics of TP SH variation. Their results indicated that for interannual and interdecadal variations of SH, the influence of the change in either density or drag coefficient is insignificant, while the main contributors are the changes in surface wind speed (*V*) and the temperature difference between the land surface and surface air (*T*_g_ − *T*_a_). Liu *et al*. [37] designed a parameter of SH, *V* (*T*_g_ − *T*_a_), to investigate the interannual and interdecadal variations of SH over the TP. As shown in Fig. 1d, the interannual variation of (*T*_g_ − *T*_a_) is comparable with that of *V*, whereas the relative change in *V* is larger than that in (*T*_g_ − *T*_a_) on the interdecadal timescale. During the analysis period, *T*_g_, *T*_a_ and (*T*_g_ − *T*_a_) all increased, whereas *V* decreased from 1980 to the end of the previous century but then increased after 2003. Consequently, the parameterized SH over the TP presents a weakened trend in summer before the end of the previous century but an increased trend since the beginning of this century. This could exert strong impact on the climate variation in surrounding areas, as shown in the following.

### AHS over the TP and associated circulation in winter and summer

**Figure 2.**
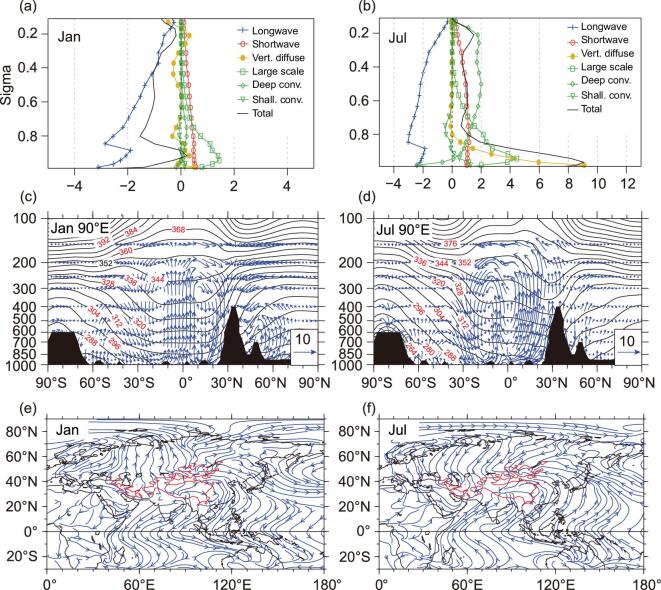
Tibetan Plateau SH-driven air pump: (a and b) vertical profiles of different components of heating rate (unit: K day^−1^) averaged over the TP (20°–40°N, 70°–100°E) with elevation above 1.5 km; (c and d) cross sections along 90°E of monthly mean potential temperature (unit: K) and circulation (vectors, unit: m s^−1^) projected onto the cross sections, and (e and f) deviations from the annual mean of monthly mean streamlines at 10 m above the surface. Data are from the NCEPII reanalysis for the period 1979–2014. The left column is for January and the right column is for July. The red contours in (e) and (f) indicate the 1.5-km elevation of the TP (refer to [10]).

Figure 1c shows that the AHS over the TP is cooling (heating) in winter (summer). The corresponding vertical profiles of the varied heating also present strong seasonal contrast. In winter, the SH, LH and shortwave radiation heating over the TP are all weak and they cannot compensate the longwave radiation cooling throughout the troposphere (Fig. 2a). The profile of total AHS indicates the existence of a remarkable inversion below the height σ = 0.85. In summer, all types of heating develop strongly in different layers of the troposphere with the strongest SH of approximately 9 K day^−1^ near the surface and their sum far exceeds the longwave radiation cooling in the troposphere (Fig. 2b).

The cooling in winter and heating in summer over the broad scale of the TP as well as that of the continents, exert a strong impact on atmospheric temperature and circulation. Cross sections of the monthly mean potential temperature and meridional circulation along 90°E in January and July (Fig. 2c and d) show cold (warm) temperatures over continental areas and warm (cold) temperatures over oceans in January (July). In January, the strongest cooling and strongest air descent occur over the TP and to its south (Fig. 2c). In July, the warmest potential temperature is just above the TP, consistent with previous findings [29]. Strong ascent prevails from the southern TP to the tropical Indian Ocean (Fig. 2d). Seasonal changes in the thermal contrast between land and oceans, and between the two hemispheres, drive surface movement of air from the winter hemisphere into the summer hemisphere, and from the continents to the oceans in winter but from the oceans to the continents in summer (Fig. 2e and f). A prominent reversal of the monthly mean circulation deviated from the annual mean occurs over the TP area. In January, surface air is pumped away from the TP toward Africa and the Southern Hemisphere (Fig. 2e), whereas the opposite occurs in July (Fig. 2f), signifying the seasonal reversal of the Asian monsoonal flows. The process repeats annually and appears as a gigantic air pump. As this air pump is driven primarily by the SH of the TP [10,38], it has been defined as the TP SH-driven air pump. In addition to the large-scale land–sea thermal contrast, the TP thermal pumping exerts strong influence on the onset, maintenance and seasonal evolution of the ASM.

## TP FORCING AND ASM

### TP forcing and ASM onset

The onset of the ASM is affected by the TP [39]. One of the key dynamic processes for monsoon onset is a reduction of upper-level absolute vorticity [40,41]. Stationary planetary waves are able to produce an intensified thermally direct circulation over areas with low absolute vorticity [42]. Springtime heating over the continent and the TP can force a stationary wave and ascent over the plateau, forming divergence collocated with non-negligible absolute vorticity off the equator and decreasing the upper-level vorticity. Thus, the lower-latitude circulation is rapidly adjusted into a thermally direct regime with an intensified monsoonal-type meridional circulation located to the south of the TP, leading to abrupt monsoon onset [43]. The earliest ASM onset occurs over the eastern Bay of Bengal (BOB) [44]. Numerical experiments have revealed that the location of this monsoon onset is anchored by TP forcing, that is moving the ‘TP’ westward by 30° longitude leads to a similar westward displacement of the location of the earliest ASM onset [45].

Previous studies have also revealed that TP forcing in spring creates favorable conditions of coupling between the upper and lower circulations for convection development and ASM onset over the eastern BOB. In spring, the subtropical westerly flow over Asia remains strong and it impinges directly on the TP in the mid–lower troposphere, forming a stationary circulation dipole over Asia with an anticyclonic (a cyclonic) gyre to the north (south) of the TP [10,46]. The southwesterly flow in front of the cyclonic gyre transports ample water vapor from the BOB to the Indochina Peninsula, causing frequent spring rainfall and leading to northwestward movement of the SAH with its center over the northern Indochina Peninsula. Thus, strong divergent flow develops over the southeastern BOB, reducing the local vorticity aloft and forming upper-layer pumping [47].

In the lower troposphere, a cold northwesterly flow along the TP-induced cyclonic gyre sweeps over India where the surface temperature is high. Strong SH is generated, forming a prominent surface cyclonic circulation over the Indian Peninsula with a strong southwesterly coastal flow over the northwestern BOB. Strong air–sea interaction is thus triggered in the BOB, forming a unique springtime warm pool with sea surface temperature (SST) of >31°C in the BOB and stimulating the development of strong convection over the south of the warm pool [48]. When this lower-layer convection is coupled with the vorticity minimum aloft and the upper-layer pumping from the SAH, the ASM starts to erupt over the southeastern BOB. Therefore, it is the TP-induced local air–sea interaction and the land–sea thermal contrast over South Asia in spring that causes the earliest ASM onset over the southeastern BOB.

### Two types of heating and the Tibetan–Iranian Plateau coupling system

**Figure 3.**
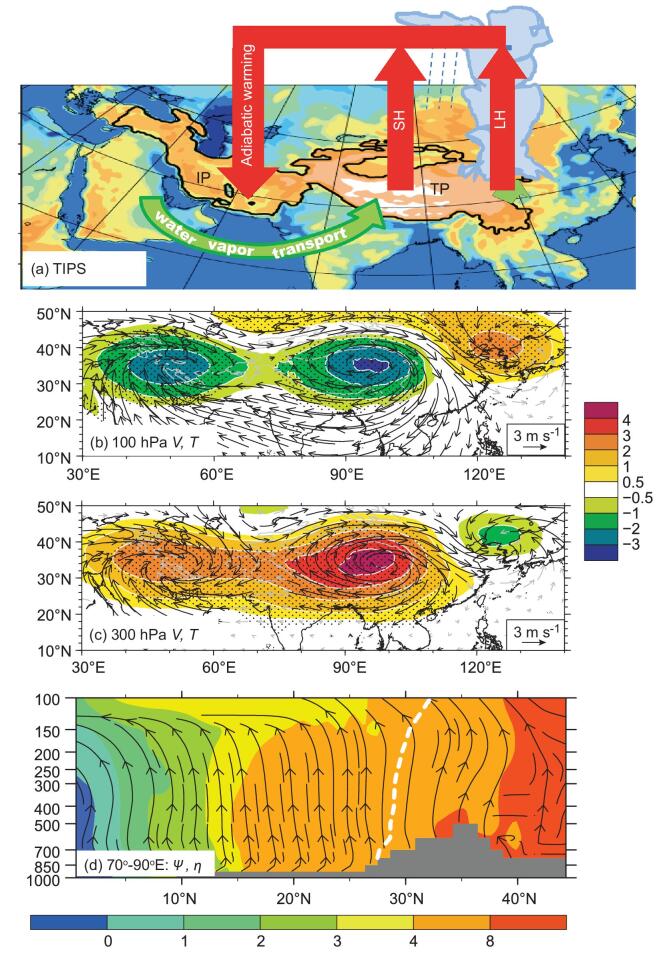
(a) Schematic of the TIPS feedback coupling system comprising the thermal forcing over both the TP and the Iranian Plateau and the water vapor transport over South Asia. (b) Differences in temperature (shading, unit: °C), wind field (vectors, unit: m s^−1^) at 100 hPa between the control experiment and the experiment without sensible heating over the TIP. Stippling indicates where the temperature difference exceeds 95% significance level; and wind difference significant above the 95% level is plotted in black. (c) As for (b) except for 300 hPa; and (d) cross sections of the July mean meridional circulation (streamlines), absolute vorticity (shading, unit: 10^−5^ s^−1^), and zero-zonal wind curve (white dashed line) for the Asian monsoon area (70°–90°E) [50,51].

Similar to the TP, the thermal forcing of the Iranian Plateau can generate a cyclonic circulation in the lower troposphere, which conveys water vapor from the Arabian Sea to India and the TP, contributing to regional rainfall. Thereby, the SH increase over the Iranian Plateau can lead to SH decrease and LH increase over the Tibetan Plateau [49]. In return, the SH increase/decrease over the Tibetan Plateau can intensify/weaken sinking motions to its west, causing SH increase/decrease over the Iranian Plateau. Thus, a quasi-equilibrium state between the SH and LH over the Tibetan Plateau, vertical atmospheric motion over the TP and the Iranian Plateau, and SH over the Iranian Plateau is reached. This so-called Tibetan–Iranian Plateau coupling system (TIPS) (Fig. 3a) can significantly influence atmospheric circulation [50]. It has been demonstrated that interaction between the SH and LH over the TP plays a leading role in the TIPS. The impacts of Iranian Plateau SH and TP SH on the climate of other regions can reinforce or offset each other. Their combined influence signifies the dominant regional water vapor transport. The heating of the TIPS warms the atmosphere aloft, elevates the tropopause and enhances anticyclonic circulation in the upper troposphere with two anomalous centers over the Tibetan–Iranian Plateau (TIP) (Fig. 3b and c). The anomalous centers are warm in the upper troposphere but cold in the lower stratosphere, leading to development of minimum absolute vorticity near the tropopause over the plateaus [51]. Subject to angular momentum conservation [52], a monsoon-type meridional circulation is generated with its descending branch in the Southern Hemisphere and its ascending branch over the southern TIP. Easterly vertical shear and large-scale ascent thus develop over South Asia (Fig. 3d), creating a favorable background for ASM development.

### Impacts of TP heating on ASM precipitation

The TP can influence the ASM through air–sea interaction, mid-latitude wave propagation and even aerosol forcings [53–55]. The surface heating effect of the TP is a crucial physical process in controlling the relationship of the TP and other weather and climate systems [56–59]. It has been shown that heating over the TP correlates well with Indian summer monsoon rainfall in the early (20 May to 15 June) and late (1 September to 15 October) monsoon season [60]. This together with the El Niño–Southern Oscillation (ENSO) explains a substantial portion of the interannual variability in early and late seasonal rainfall, and it provides potential for predictability.

**Figure 4.**
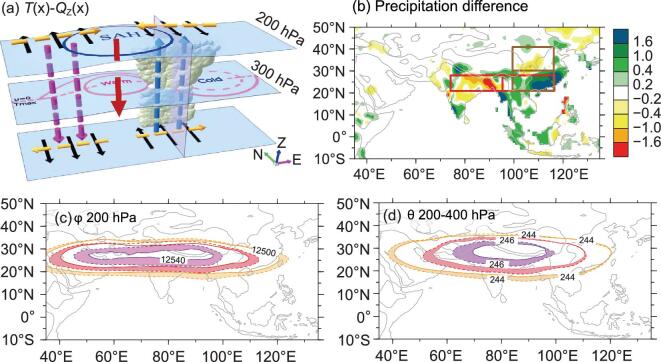
(a) Schematic of the *T-Q_Z_* mechanism. The warm center and SAH in the upper troposphere present on the eastern end of the cooling and to the west of the heating (blue arrow, monsoon heating; purple arrow, vertical descent; black arrow, vertical shear of meridional wind; and brown arrow, Coriolis force); and decadal changes in JJA mean climate between the periods of 1991–2000 and 1981–1990 of (b) precipitation based on the PREC/L dataset (unit: mm d^−1^), (c) 200-hPa geopotential height (unit: gpm), and (d) 200–400 hPa mass-weighted temperature (unit: K) based on ERA40 reanalysis data [63]. The two pairs of boxes in (b) indicate the south–north seesaw and the west–east seesaw.

The TP thermal impact on the ASM can also be detected on the decadal timescale. As shown in Fig. 1d, the SH of the TP has declined from the mid-1970s to the end of the 20th century. Data diagnosis and numerical experiments [37,61] have demonstrated that reduction of the SH of the TP in spring and summer weakens the forced near-surface cyclonic circulation. Consequently, the summertime southerlies that usually prevail over eastern China are weakened, and the rain belt associated with the ASM remains over South China, instead of moving northward. The distribution of precipitation difference between the last and second last decades of the 20th century is shown in Fig. 4b. An apparent anomalous pattern can be seen with enhanced rainfall over South China and reduced rainfall over North China, presenting a north–south seesaw axis over East China (see the two brown boxes in Fig. 4b), that is the so-called ‘South flood–North drought’ pattern.

For a steady-state frictionless atmosphere in the subtropics, the meridional wind *v* and the vertical gradient of diabatic heating *Q* can be well expressed as in the following Sverdrup balance [62]:

$$\begin{eqnarray*}\beta \nu \&\approx\& (f + \zeta )\theta _z^{ - 1}(\partial Q/\partial z),\nonumber\\ \&{\theta _z}\ne\& 0,{\rm{\vec{V}}} \cdot \nabla \zeta \to 0.\end{eqnarray*}$$

Following the thermal wind relation between temperature and meridional wind *v*, and assuming normal distributions for both diabatic heating *Q* and temperature *T*, then *T* and *Q* approximately satisfy the following relation [63]:

$$\begin{equation*}T(x) \approx {\lambda ^2}(\partial Q(x)/\partial x),\end{equation*}$$

where ${\lambda ^2}$ is a positive coefficient. It implies that the heating-induced temperature anomaly *T*(x) lags the heating *Q*(x) by one-quarter phase, as shown schematically in Fig. 4a. Thus, increased convectional heating can enhance the upper-tropospheric warm center together with the SAH to the west, intensifying the sinking motion further to the west. This agrees well with the observed climatic mean longitudinal distributions of temperature and diabatic heating along the subtropics in the upper troposphere [63]. During the final two decades of the 20th century, as the SH over the TP decreased (Fig. 1d) and the precipitation over South China increased (Fig. 4b), the SAH and the upper-tropospheric warm center intensified (Fig. 4c and d), while precipitation over North India and Bangladesh decreased (Fig. 4b). Namely, the rainfall anomaly presents a west–east seesaw along the Asian subtropics (see the two red boxes in Fig. 4b). The results demonstrate that anomalous heating over the TP exerts an important impact on the configuration of climate anomalies, at least over the ASM area.

### Debate on the maintenance of the South Asian summer monsoon

In a recent model experiment in which the main part of the TP was removed but the Himalayas were retained, the simulated South Asian summer monsoon was found to be largely unaffected and thus a new hypothesis was proposed. This hypothesis considers the Himalayas as a thermal isolator that prohibits the cold dry northerly flow (with low entropy) from the mid-latitudes, which means the high entropy air in the lower layers over India can be coupled with the warm center in the upper troposphere through monsoonal convection over South Asia [64, hereafter BK10; 65]. This new hypothesis has stimulated debate on the maintenance of the South Asian summer monsoon [66–69], which has advanced the overall understanding of the relevant science.

The barrier hypothesis was reexamined by conducting a series of independent numerical experiments that included full TP, barrier and no-TP configurations [70]. The proposed barrier-induced blocking mechanism was not found and the sensible heating on the slopes of the TP was considered a driver of the South Asian monsoon. It transpires that the key is the surface sensible heating on the slopes of the Himalayas, which was retained in BK10’s experimental design such that the thermal pumping effect of the TP was included. Once the surface sensible heating on the slopes of the Himalayas is removed, the northern branches of the South Asian summer monsoon and the East Asian summer monsoon disappear [69]. Physically, the surface insolation in summer is higher to the north of the TP than to its south, and the prevailing wind in the lower troposphere across the TP is southerly because the land–sea thermal contrast in summer produces a continental-scale surface cyclonic circulation over the continent and because the TP is located in the eastern Afro-Eurasian continent [67]. Thus, there is no northerly advection, which means the presence of a thermal isolator for preventing cold dry invasion from the north is not required [68]. Second, as mentioned above (Fig. 4a), descending motion occurs under the central and western SAH and the upper-tropospheric warm center. There is no convection for coupling the high energy in the lower troposphere and the warm center above. The high surface entropy over India in summer associated with monsoon development is indeed mainly a result of its high water vapor content, which, as described above, is transported from the Arabian Sea and BOB via the TIP thermal pumping [68]. The ASM is in fact controlled thermally by both the land–sea contrast and the elevated heating of the TP [69].

The above does not mean that the mechanical barrier effect is unimportant. This effect in winter not only splits the impinging westerly flow into northern and southern branches but also blocks the northerly flow in the lower troposphere. The cold intrusion into India thus becomes a deflected northwesterly or northeasterly flow [68]. In summer, the TP barrier effect causes the prevailing southerly flow to climb its southern slope, forming heavier precipitation when there is surface heating on the sloped surface. If there were no surface heating, the barrier effect would cause the impinging southerly flow to deflect around the TP and thus there would be no monsoon over northern India [67].

## IMPACTS OF THE TP ON UPSTREAM CLIMATE OVER WEST ASIA, EUROPE, AFRICA AND THE NORTH ATLANTIC

The climate over Asia and Africa/Europe is strongly linked by zonal–vertical overturning circulation in the lower latitudes and wave-train patterns in the higher latitudes, which are related to divergent and rotational portions of the atmospheric motion, respectively. While the latitudinal heating gradients between warmer continents and cooler oceans has been recognized as the dominant forcing of ASM formation and development since the time of Halley [71], longitudinal heating gradients were also believed important for the monsoon [72–76]. Given that large latent heating occurs in the monsoon region and radiative cooling is dominant in the African desert region, and given that the longitudinal gradient of total heating between Asia and North Africa in summer is of the same order of magnitude as the latitudinal gradient across the Asian monsoon region [76,77–79], it has been hypothesized that the longitudinal heating gradient is a potential factor that influences ASM variability.

Although the increase in anthropogenic aerosols and greenhouse gases might play a role in the drying trend of Sahel rainfall since the 1950s [80–83], a recent study by He *et al*. [84] revealed that the strengthened latent heating associated with the ASM could explain the long-term decrease in Sahel rainfall through an anomalous zonal–vertical circulation. The robustness of this feature can be demonstrated by model experiments with enhanced heating over either South Asia or Southeast Asia. More recently, Sy *et al*. [85] related the climate link between South Asia and the Sahel to variation of the tropical easterly jet stream in the upper troposphere.

In higher latitudes, especially during the cold seasons, the interaction between the TP and wave-train patterns, including Rossby wave propagation, is an issue of considerable interest. Within this context, both the influence of atmospheric teleconnection patterns on the climate over the TP and adjacent regions [10] and the impact of the ASM on the high-latitude upstream climate have attracted certain research interest. For example, Rodwell and Hoskins [86,87] claimed that the characteristic Mediterranean climate was a Rossby-type response to Indian summer monsoon heating, while Zhao *et al*. [88] found that the origin of summer climate signals over the North Atlantic and European regions could be traced to the Asian continent. The effects of large-scale orography on the Northern Hemisphere climate including the monsoonal climate and mid-latitude dry climate have been discussed widely [89–91]. The dry climate over central Asia is well reproduced in model experiments with mountains because of the stationary waves during the cold seasons and the South Asian monsoon circulation during summer that are induced particularly by the TP. In contrast, a moist climate appears in experiments without mountains, indicating the crucial role of the TP in formation of the central Asian climate [90].

It is important to consider the role that the TP might have in the connection between the plateau and the upstream climate or its impact on the upstream climatic variation. Model experiments have demonstrated that the TP plays an important role in modulating both the downstream East Asian monsoon and the upstream desert climate [92]. TP heating also drives the variability of Pakistan monsoon rainfall via a TP-induced Rossby wave response in the upper troposphere and weakened water vapor transport from the BOB to Pakistan [19]. Furthermore, a significant positive relationship exists between the tropospheric temperature over the TP and rainfall over the central–eastern Sahel during summer. This is manifest through an anomalous zonal–vertical cell with ascent over the TP and descent over the Mediterranean, as well as an anomalous meridional cell with rising motion over the Sahel [18].

**Figure 5.**
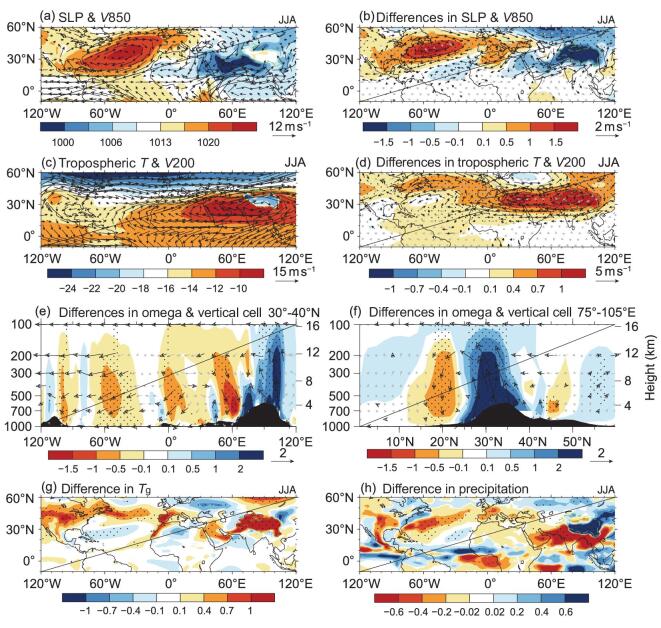
JJA climatology of (a) sea level pressure (shading, unit: hPa) and 850-hPa wind (vectors, unit: m s^−1^), (c) tropospheric temperature (shading, unit: °C, averaged from 700 to 200 hPa where the terrain is <3000 m and from 500 to 200 hPa where the terrain is >3000 m) and 200-hPa winds (vectors, unit: m s^−1^) in the control experiment. (b) and (d) Corresponding differences between the TP heating and control experiments, respectively. The differences between the TP heating and control experiments are shown in (e) for 30°–40°N averaged omega (shading, unit: Pa s^−1^, multiplied by -100) and zonal–vertical circulation (vectors; zonal wind in m s^−1^, and omega in Pa s^−1^, multiplied by -100), and in (f) for 75°–105°E averaged omega (shading, unit: Pa s^−1^, multiplied by -100) and meridional–vertical circulation (vectors; meridional winds in m s^−1^, and omega in Pa s^−1^, multiplied by -100). Differences are also shown in (g) for surface temperature (unit: °C) and in (h) for precipitation (unit: mm d^−1^). Dots indicate the values that significantly exceed the 95% confidence level. In (b), (d), (e) and (f), the values of wind differences significantly above the 95% confidence level are plotted in black vectors. Dashed green lines indicate the TP region where the terrain is >1500 m. Black shading represents terrain height.

**Figure 6.**
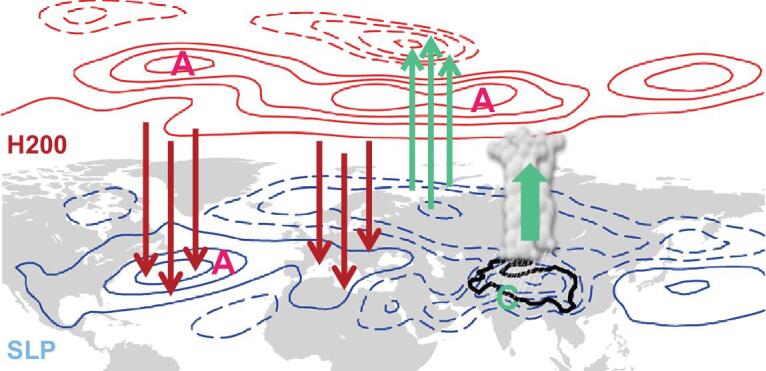
Schematic of the impact of TP heating on upstream climate. Red contours indicate the changes in 200-hPa geopotential height and blue contours denote changes in sea level pressure (solid contours, positive anomalies; dashed contours, negative anomalies). A (C) indicates anticyclonic (cyclonic) anomalies. Green (red) vectors represent upward (downward) anomalies.

A series of experiments with earth system models has illustrated that TP heating exerts strong impact on the climate over West Asia, the Middle East, North Africa, South Europe and the North Atlantic [16,18]. Figure 5 presents the changes in horizontal circulation, tropospheric temperature and vertical circulation associated with heating of the TP surface from surface albedo reduction. According to the so-called SH-driven air pump effect [10,93], TP heating leads to increases in regional tropospheric temperature and thickness of the air column (Fig. 5c and d). In the upper troposphere, the SAH intensifies and extends westward, and to the west of the TP, a distinct Rossby wave response emerges with anomalous anticyclonic circulation and warming throughout the entire troposphere. In the lower–middle troposphere, the Atlantic subtropical high intensifies and moves northwestward (Fig. 5a and b), accompanied by northward shifts of the Atlantic Intertropical Convergence Zone (Fig. 5h) and the local Hadley circulation (not shown). Moreover, features of a thermally driven circulation are apparent (Fig. 5e and f). An anomalous zonal–vertical cell appears with an ascending branch over the TP and a descending branch over the Mediterranean Sea, and an anomalous meridional–vertical cell occurs with an ascending branch over the TP and a descending branch over India and adjacent regions. Therefore, subsidence strengthens over the Mediterranean and the North Atlantic, decreasing rainfall (Fig. 5h) and increasing surface temperature correspondingly (Fig. 5g). In short, as depicted by the schematic in Fig. 6, warming, anomalous ascent, a low-level cyclone and a high-level anticyclone occur when heating increases over the TP, inducing anticyclonic anomalies (intensified SAH and Atlantic subtropical high), reduced rainfall and warming over the Mediterranean Sea and extratropical North Atlantic through both the Rossby wave response and the thermally driven vertical circulation. Within this framework, changes in the tropical divergent part and the extratropical rotational portion of the atmospheric circulation related to TP heating are emphasized. Limited evidence indicates that the effect of the northern portion of the TP relative to its southern counterpart favors a circumglobal pattern that affects the climate of the North Atlantic, South Europe and North Africa (figure not shown).

It is also interesting to elucidate the relative importance of TP heating to Asian continental heating regarding modification of the upstream climate variations. Based on the relative changes in circulation patterns, surface temperature and rainfall, TP heating accounts for approximately 40–50% of the climate signals induced by Asian continent heating, although the TP domain occupies only one-third of the area of the Asian continent according to numerical model experiments [16].

Given the interactive nature between SST and its overlying atmosphere, the North Atlantic SST signals caused by the thermal impact of the TP in turn influence the variations of upstream climate. The feedback of these SST signals accounts for above 20% of the total upstream signals caused by TP heating, as assessed by numerical model experiments with and without Atlantic SST variations [17]. Compared with experiments in which TP heating is included but Atlantic SST variation is excluded, an anomalous wave pattern characterized by three positive centers (over the extratropical North Atlantic, Arctic Ocean and to the east of Japan) and four negative centers (over the central North Atlantic, North Europe, northeastern North America and northwestern Pacific) appears when Atlantic SST variation is involved. Corresponding to the circulation pattern, rainfall increases over northeastern North America and North Europe but decreases over the northwestern Atlantic. Because of the reduced strength of the westerlies related to the reduced strength of the thermal low over subtropical Africa, the increase in Sahel rainfall is less evident when the change in Atlantic SST is considered (Fig. 5h).

Previous studies have also revealed that the heat source over the TP can be affected by early spring SST anomalies over the North Atlantic through a stationary wave train that crosses the North Atlantic, North Europe and the TP, and then modulates the subtropical westerly jet stream [94]. Given the various timescales and spatial domains of Atlantic SST variation, the impact of the TP on upstream regions is much more complex when the feedback of different types of Atlantic SST variation is considered.

## TP EFFECT ON THE AMOC

The rapid uplift of the TP began approximately 50 Ma (million years ago) and was accelerated at approximately 10–8 Ma [95,96]. It triggered establishment of the Indian and East Asian monsoons at around 9–8 Ma, resulting in enhanced aridity in the Eurasian interior and an intensified westerly jet stream in winter in the Northern Hemisphere [97–99], as well as increased dust transport to the North Pacific Ocean [99]. These features could have resulted in freshening and cooling of the North Pacific and weakening of the Pacific meridional overturning circulation [100]. However, the timing of significant uplift of the TP was close to that of the establishment of the AMOC [101, and references therein]. In contrast, the Rocky Mountains were fully developed by 45 Ma, far earlier than the establishment of the AMOC. Paleoclimatic evidence implies an important role of the TP in the formation of modern thermohaline circulations.

The AMOC, one of the key elements of the global climate system, is commonly recognized as being sustained by North Atlantic deep-water formation (NADW) on short timescales [102–104]. Considering both the thermal and the mechanical effects of the TP, Fallah *et al*. [22] conducted topography-sensitive experiments with a coupled GCM (CGCM) and found that the TP could significantly affect both the North Atlantic SST and the AMOC. Recent investigations into the effect of the TP on the AMOC using the fully coupled climate model, demonstrated that removing the TP would result in collapse of the AMOC [105].

**Figure 7.**
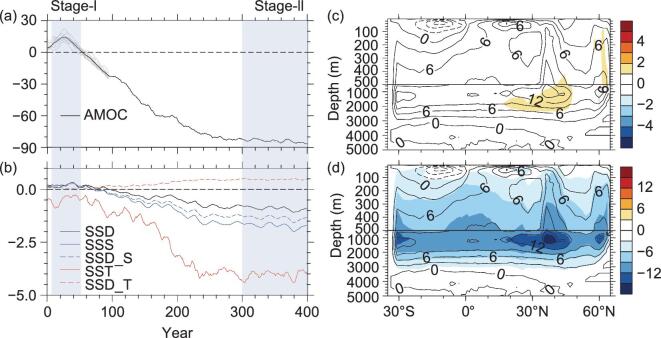
AMOC index and patterns. (a) Percentage change in the AMOC index (black curve), which is defined as the maximum streamfunction in the range of 0°–10°C isothermal between 20°N and 70°N in the Atlantic. Gray curves show the AMOC changes in 10 ensemble runs. (b) Changes in sea surface salinity (SSS; blue, unit: psu), temperature (SST; red, unit: °C), and density (SSD; black, unit: kg m^−3^), SSS-induced SSD change (dashed blue) and SST-induced SSD change (dashed red). In (b), all variables are averaged over the region 40°–65°N, 10°–60°W and 0–30 m. (c) and (d) Mean AMOC in control experiment (Real) (contour, unit: Sv, 1 Sv = 10^6^ m^3^ s^−1^) and its change (shading, unit: Sv) in Stage-I and Stage-II, respectively, which are defined in (a). All changes are obtained from TP removal experiments (NoTibet), with respect to Real (adapted from [105]).

Figure 7 shows the changes in the AMOC index and its pattern in response to TP removal. The AMOC is enhanced by as much as 20% in the first few decades of model integration and then weakened by more than 80% after 300 years (black curve, Fig. 7a), which is a feature confirmed by 10 ensemble runs (gray curves, Fig. 7a). The experimental result implies the importance of the TP in the establishment of the AMOC in the modern climate. The quasi-equilibrium response of the AMOC (year 300–400) is comparable with that in experiments when freshwater is hosed into the North Atlantic [106–108]. The spatial patterns of AMOC changes in the initial stage (Stage-I) and the quasi-equilibrium stage (Stage-II) are plotted in Fig. 7c and d, respectively. The patterns exhibit a marginally positive anomaly in Stage-I (Fig. 7c) and a significant reduction of downward mass transport in the subpolar North Atlantic in Stage-II, that is collapse of the AMOC (Fig. 7c and d). The AMOC comprises a wind-driven circulation and a thermohaline circulation [109]. The wind-driven circulation in the tropics is affected minimally by TP removal (Fig. 7c and d); thus, the major influence of the TP on the AMOC is from the thermohaline branch.

Surface buoyancy change in the NADW region (40°–65°N, 10°–60°W) is the key factor determining the response of the AMOC [110]. The sea surface density (SSD) averaged in the NADW region increases slightly during the first 50 years and then decreases remarkably subsequently (Fig. 7b), consistent with the evolution of the AMOC. The SSD change can be further split into the change induced by SST and that attributable to sea surface salinity (SSS). The North Atlantic SST is cooled during the entire 400 years (red solid line), which increases SSD (red dashed line). However, SSS increases during the first few decades and then decreases during the remainder of the integration (blue solid line), driving SSD changes accordingly (blue dashed line). In general, the SSD increase during Stage-I leads to a stronger AMOC, of which 30% is attributable to surface cooling and 70% to surface salinization, while the SSD decrease later on is exclusively attributed to surface freshening, resulting in the collapse of the AMOC.

**Figure 8.**
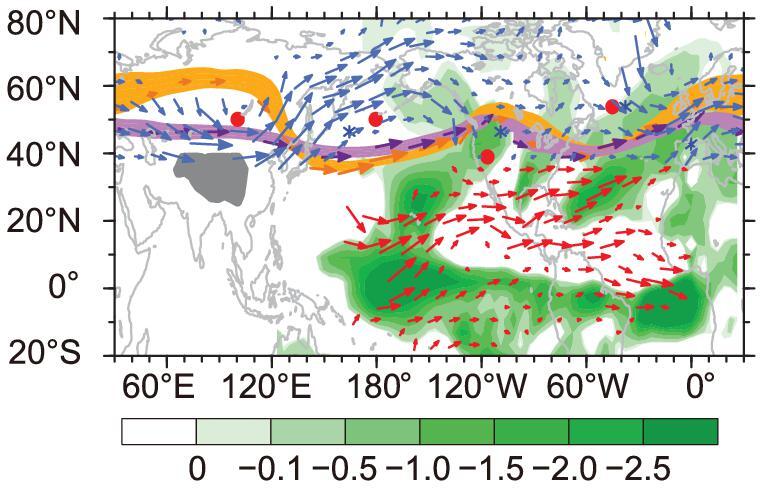
Wave train, westerlies and water vapor transport. Brown ribbon and arrows show schematically the stationary wave structure and corresponding winds at 850-hPa geopotential height in Real. Purple ribbon and arrows are the same as the brown ones, except for NoTibet. Red dots (blue stars) represent the high- and low-pressure centers in Real (NoTibet). Blue arrows are the wind changes in NoTibet with respect to Real, showing enhanced westerlies. Red arrows represent the change in vertically-integrated water vapor transport in NoTibet with respect to Real. Green shading represents the change in water vapor convergence in NoTibet, and negative values mean that the ocean gains freshwater from the atmosphere. The TP is indicated by the gray patch.

The mechanism via which the TP affects the AMOC presented by Yang and Wen [105] is summarized in Fig. 8. Removing the TP would intensify the westerlies over the North Atlantic, enhancing ocean surface evaporation that would result in greater heat loss to the atmosphere through LH and SH fluxes, and strengthen the southward Ekman flow, which would bring additional cold water from the Greenland–Iceland–Norwegian seas into the North Atlantic. Atmospheric processes are vital in Stage-I. SSS increase in the first few decades after TP removal is attributed to the intensified westerlies that enhance evaporation and cold-water advection from the north. However, the subsequent SSS decrease is because of greater freshwater transport from the tropical Pacific to the North Atlantic along the so-called atmospheric river [111], converging over the North Atlantic and leading to freshening of the upper ocean (red vectors and green shading, Fig. 8). This surface freshening weakens the NADW, triggering a decline of AMOC. The teleconnection pattern shown in Fig. 8 is established mainly via atmospheric processes [112–115]. Oceanic processes do not contribute to the teleconnection; instead, they respond to the teleconnection. In other words, the TP affects the oceanic circulation and buoyancy fields via atmospheric processes [22].

The modeling results of Yang and Wen [105] are qualitatively consistent with those of previous studies by Fallah *et al*. [22], Maffre *et al*. [23] and Su *et al*. [116], that is removal of the TP results in collapse of the AMOC. However, the detailed processes that lead to AMOC collapse are different in different studies. For example, Fallah *et al*. [22] emphasized the role of reduced northeastward heat advection from the North Atlantic in weakening of the AMOC. Maffre *et al*. [23] concluded that the westward freshwater transfer across Africa is critical to the freshening of the Atlantic and thus to the weakening of the AMOC. Su *et al*. [116] identified the critical factor as northward moisture transport over the North Atlantic. However, these studies did not show the transient change in the AMOC. The study of Yang and Wen [105] showed initial strengthening of the AMOC in response to TP removal, followed by subsequent weakening. Moreover, they emphasized that the atmospheric moisture relocation from the tropical Pacific to the North Atlantic is the key that triggers the weakening of the AMOC, and the positive feedback between the southward expansion of sea ice and the AMOC decline leads to the AMOC shutdown eventually. These mechanisms are different from those discussed in previous studies. The discrepancies in the mechanisms reflect the complexity of the effect of the TP on global ocean circulations. Consequently, further sensitivity experiments using different models are needed.

## TP AND OCEANS: SYNERGISTIC CLIMATE INFLUENCE

Although the TP and its thermal status are treated as a forcing source for generating regional and remote anomalies in atmospheric and oceanic circulations, the TP forcing itself should be considered a result of atmospheric and oceanic circulations. This is because the heating source and mechanical forcing of the TP are determined by various atmospheric variables such as wind, temperature, humidity and ground surface heat flux. Revealing how TP forcing is manifest could help elucidate the role of TP forcing in the framework of land–air–sea interactions. In recent decades, the interaction between the TP and oceans, together with their synergistic role regarding the Asian monsoon, has drawn increasing attention. The consensus is that the TP can interact with remote oceans through westerly jet streams, Rossby waves, local atmospheric circulations and teleconnections. Here, two aspects are discussed: the interaction between the TP and the oceans, and their synergistic influence on the East Asian summer monsoon.

The persuasive research method adopted to clarify the impact of the TP on oceans is to use climatic models CGCMs (e.g. [8,117–119]) and to conduct experiments with different degrees of global terrain elevation. It was found that the Asian monsoon climate and Indo-Pacific SSTs respond significantly to TP forcing via changes in cloud radiation and wind evaporation processes. Because the Pacific Ocean is located downstream of TP along the westerly belt, the *in situ* SST anomaly (SSTA) is inevitably related to TP thermal forcing. Zhao *et al*. [115] found that the atmospheric circulations over the TP and the North Pacific are closely related and hence, they proposed the concept of the Asia Pacific Oscillation. Nan *et al*. [120] further argued that the tropospheric temperature over the TP might induce a warm SSTA in the equatorial central–eastern Pacific through the Asia Pacific Oscillation.

**Figure 9.**
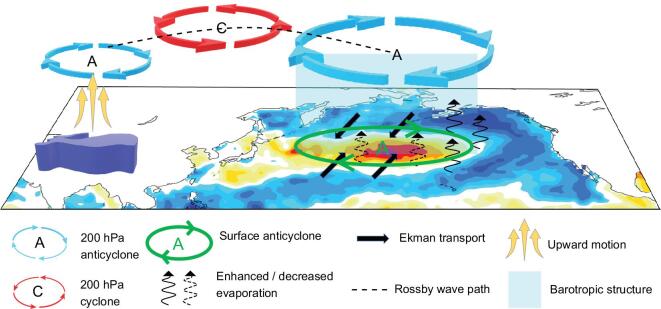
Schematic showing the role of spring TP heating in alternating North Pacific SSTA.

As mentioned above, the AHS over the TP in spring is dominated by SH. Based on GCM experiments, Duan *et al*. [121] found that TP heating could affect the western Pacific subtropical high by changing the equatorial Pacific SST. Sun *et al*. [122] also indicated that TP SH in spring could affect the North Pacific by generating an anomalous barotropic atmospheric circulation. As a result, the underlying SST is altered significantly through sea surface heat exchange and Ekman transport (Fig. 9).

In addition to the significant impacts of the TP on North Atlantic SST, SSS and the AMOC, previous studies have also focused on the thermal effect of the TP on air–sea interaction in the Indian Ocean, especially in summer. Wang *et al*. [123] employed a regional climate model to examine the relationship between the thermodynamic processes of the TP in summer and air–sea interaction in the Indian Ocean, and indicated that TP thermal forcing could obviously reduce the northern Indian Ocean SST by enhancing southwesterly winds. Meanwhile, monsoonal convective activity around the regions of decreased SST in the northern Indian Ocean was suppressed. A similar result was reported by He *et al*. [124] following comparison of atmospheric GCM and CGCM experiments. It is further concluded that air–sea interaction processes can somehow offset the impact of TIP thermal forcing on the surrounding atmospheric circulation, and that upwelling (downwelling) motion in the east (west) along the equatorial Indian Ocean can be detected from CGCM experiments. Almost at the same time, another independent work by Baldwin *et al*. [125] showed that with TP topography included in a CGCM, the northwestern Pacific SST would respond as a dipole pattern and upwelling would be enhanced in the Arabian Sea, resulting in local SST cooling. All these results indicate the non-negligible role of large-scale topography in the regulation of global SSTs and ocean currents.

In addition to the remote influence of the TP, remote forcing from global oceans on the thermal conditions of the TP has also been emphasized in recent years. In the mid–high latitudes, the contributions from atmospheric internal modes, for example the North Atlantic Oscillation, and oceanic variability such as the North Atlantic Tripolar SST pattern, on the interannual variation of TP diabatic heating have been revealed ([94,126]). On longer timescales, Shi *et al*. [127] found that convection (temperature) over the plateau was suppressed (increased) during the warm phase of the Atlantic multidecadal Oscillation.

It is noteworthy that the relationship between TP thermal forcing and global and regional SSTAs exhibits strong seasonality. In early spring, the general circulation over the TP and Asian monsoon regions is still in the winter state and the AHS over the TP is influenced mainly by the mid–high-latitude circulation in the Northern Hemisphere. After the onset of the Southeast Asian monsoon in early May, accompanied by weakening and northward retreat of the westerly jet stream, the relationship between the tropical oceans, especially the Indian Ocean, and the TP diabatic heating shows a more intimate interconnection. Specifically, the Indian Ocean Basin Mode (IOBM) can significantly affect the heating condition of the TP by altering the local meridional circulation and suppressing or enhancing ascent over the TP [35]. In terms of a pure Indian Ocean Dipole, through response of the barotropic Rossby wave, the negative geopotential height over northern India can induce warm humid southwesterly flows that benefit the snow cover over the TP. In contrast, the anomalies of moisture are insignificant in relation to a pure El Niño effect. However, there is significant positive partial correlation between TP snow cover in the preceding spring/summer and ENSO at the end of the year [128]. Overall, both tropical and mid-latitude ocean signals can affect the thermal conditions of the TP, but their relative importance and the influencing pathways depend on timescales and seasonal phases.

The interannual variability of the East Asian Summer Monsoon (EASM) is controlled by atmospheric internal variability and external forcing. Through data diagnosis and numerical simulations, the relative importance of the thermal forcing of the TP and the first leading mode of the Indian Ocean SSTA, that is the IOBM, with regard to the interannual variability of the EASM circulation was investigated [129]. Results demonstrated that stronger thermal forcing of the TP could enhance the SAH in the upper troposphere, strengthen the southwesterly (northerly) wind over southern (northern) China in the lower troposphere, and hence intensify the main rainfall belt of the EASM, which normally extends from the middle–lower reaches of the Yangtze River to Japan. Conversely, the positive phase of the IOBM could drive an anticyclonic anomaly over the northwestern Pacific in the lower troposphere, consistent with the dominant mode of the EASM circulation system. Overall, the positive phase of the IOBM and the stronger AHS over the TP are both favorable for above normal EASM precipitation.

## PROSPECTS

Despite the recent advances in studies of the thermal status of the TP and of its climate impacts, many unknowns and challenges remain.

It has been demonstrated that the elevated thermal status of the TIPS exerts significant impact on atmospheric circulations and the global climate, particularly the maintenance of the ASM. However, mechanisms for generation and maintenance of the monsoon can be different from that responsible for its variation on different timescales. Given the existence of both the land–sea thermal contrast and the thermal forcing of large-scale orography in the current climate system, the relative contributions to monsoon variability of the mechanical forcing of the TP versus its thermal forcing and of the remote effects versus the local thermal impacts remain unclear and require further study.

Improved understanding of the thermal status of the TP and of its climate impact is helpful for enhancing the skill of climate prediction. Knowledge of clouds and their role in TP climate variations remains limited because of the sparse observations before the 1970s. Since 2006, with the launch of the CloudSat and CALIPSO satellites, the vertical structure of clouds and their radiative effects over the TP have been the focus of considerable research interest [130,131]. However, current climate models and even reanalysis systems still cannot simulate adequately the vertical structure of clouds [132]. To improve simulations of clouds and precipitation over the TP, additional efforts are required to reveal the roles of the physical processes of both the planetary boundary layer and the underlying surface of the TP in shaping shallow and deep convective clouds.

The previously sparse distribution of meteorological observation stations over the TIP area meant information for quantification of the land–air coupling processes over the TP was insufficient. The Chinese Meteorological Administration, together with the provinces near the plateau, has now provided 2 billion Yuan (RMB) to construct an improved observation network over the TP comprising more than 6000 automatic weather stations before 2023. This network is expected to lead to marked improvement in our understanding of the coupled land–air processes and the thermal features of the TP, as well as their variations.

Correlation diagnosis, statistical analysis and numerical modeling have all been used widely to reveal the impacts of TP forcing on downstream circulation and climate, whilst dynamic approaches are helpful for improved understanding of these impacts. From the potential vorticity–diabatic heating (PV-Q) perspective, it is known that the strong diurnal change in surface heating of the TP in summer significantly influences the land–air coupling and provides favorable background conditions for the genesis of a plateau vortex during the night [133]. In winter, the impinging westerly flow is split into northern and southern branches in the lower layer by the TP [1,2]. These branches then merge on the lee side of the TP to form remarkable convergence in the boundary layer and to generate positive PV near the surface over the eastern TP (where the elevation is near the middle of the troposphere). Eastward advection of the generated positive PV along the westerly basic flow can initiate cyclogenesis and ascent that result in anomalous circulation and climate anomalies downstream of the plateau through processes study [134,135].

Many African and European regions are highly populated and the regional ecosystems and environments are susceptible to global climate change; therefore, better understanding of the impact of the TP on its upstream climate is very important because of the strong link between the TP and its upstream regions. Although previous studies have demonstrated numerous changes in the upstream climate that appear linked to the conditions of the TP, the physical processes through which the plateau might exert its influence remain unclear. For example, in the mid–high latitudes, the rotational portion of atmospheric motion is dominant and forcing, such as that derived from the TP, usually generates wave-train patterns. Therefore, it is important to consider whether the changes in TP conditions directly ‘block’ the eastward propagating signals causing variations of the upstream climate, or generate signals that propagate eastward (and even globally) across the North Pacific and North America to affect regions to the west of the plateau. Future investigations are needed to provide answers to the many related important questions.

Understanding the modulation of the TP on air–sea interaction is challenging in the study of climate dynamics. Despite encouraging progress in exploration of the interactions between the TP and global oceans, various important issues remain unaddressed. For example, it will be important to determine how such modulation influences the variability of the ASM and the ENSO–monsoon relationship. Moreover, the question of whether the melting of Arctic sea ice is related to continuous weakening of the TP heat source is of fundamental importance. In addition, further investigation is required to establish the optimal method for measuring the relative importance of external forcing (e.g. ocean signals, the North Atlantic Oscillation and others) to the variability of the TP heat source against its self-sustained variability.
